# Modelling the pulse population-wide nucleic acid screening in mitigating and stopping COVID-19 outbreaks in China

**DOI:** 10.1186/s12879-023-08265-1

**Published:** 2023-05-03

**Authors:** Qian Li, Yao Bai, Biao Tang

**Affiliations:** 1 Department of Applied Mathematics, Xi’an University of Technology, 710048 Xi’an, People’s Republic of China; 2 Department of Infectious Disease Control and Prevention, Xi’an Center for Disease Prevention and Control, 710054 Xi’an, People’s Republic of China; 3 School of Mathematics and Statistics, Xi’an Jiaotong University, 710049 Xi’an, People’s Republic of China

**Keywords:** COVID-19, Nucleic acid screening, Runs on medical resource, Screening paradox, Mathematical model

## Abstract

**Background:**

During 2021-2022, mainland China experienced multiple times of local COVID-19 outbreaks in several cities, including Yangzhou, Xi’an etc., and the Chinese government persistently adopted the zero-COVID policy in combating with the local outbreaks.

**Methods:**

We develop a mathematical model with pulse population-wide nucleic acid screening, part of the zero-COVID policy, to reveal its role in controlling the spread of COVID-19. We calibrate the model by fitting the COVID-19 epidemic data of the local outbreaks in Yangzhou and Xi’an, China. Sensitivity analysis is conducted to investigate the impact of population-wide nucleic acid screening on controlling the outbreak of COVID-19.

**Results:**

Without the screening, the cumulative number of confirmed cases increases by $77.7\%$ and $62.2\%$ in Yangzhou and Xi’an, respectively. Meanwhile, the screening program helps to shorten the lockdown period for more than one month when we aim at controlling the cases into zero. Considering its role in mitigating the epidemics, we observe a paradox phenomenon of the screening rate in avoiding the runs on medical resource. That is, the screening will aggravate the runs on medical resource when the screening rate is small, while it helps to relieve the runs on medical resource if the screening rate is high enough. We also conclude that the screening has limited effects on mitigating the epidemics if the outbreak is in a high epidemic level or there has already been runs on medical resources. Alternatively, a smaller screening population per time with a higher screening frequency may be a better program to avoid the runs on medical resources.

**Conclusions:**

The population-wide nucleic acid screening strategy plays an important role in quickly controlling and stopping the local outbreaks under the zero-COVID policy. However, it has limited impacts and even increase the potential risk of the runs on medical resource for containing the large scale outbreaks.

## Background

An emerging coronavirus, known as “Severe Acute Respiratory Syndrome coronavirus type 2” (SARS-CoV-2) caused a world-wide coronavirus disease 2019 (COVID-19) pandemic [1, 2]. Up until October 3, 2022, the global number of confirmed cases exceeds 620 million [3]. In China, the nationwide epidemic wave centered on Wuhan, Hubei province was contained by April 2020 with strict and effective non-pharmaceutical interventions (NPIs). In the nearly two years, the Chinese government persistently adopted the zero-COVID policy in combating with the COVID-19 epidemics. Here, the center purpose of the zero-COVID policy is to control the infections into zero in a short time period with a small epidemic size [4–7]. To this end, a package of strict NPIs, including the contact distancing and quarantine strategies, also the large-scale population-wide nucleic acid screening program, are carried out. Therefore, although China experienced more than 100 independent outbreaks (with a starting time and end time of reported cases) in different cities, the implementation of zero-COVID policy has helped to control the infections into zero in almost one month, consequently there is long epidemic-free period in almost cities except the period of the local outbreaks. That is, the real epidemic data from the National Health Commission of the People’s Republic of China strongly supports the fact that the zero-COVID policy indeed achieved its goal [8]. Therefore, quantifying the role of the zero-COVID policy would be essential not only for controlling COVID-19, but also for other emerging infectious diseases.

As we mentioned above, in the zero-COVID policy in China, the population-wide nucleic acid testing is one of the key control interventions. Here, the population wide testing aims at testing the whole population in the city with epidemic every few days (like two or three days), where “ten-in-one” or “twenty-in-one” pooled nucleic acid screening test method is adapted [9, 10]. The mentioned pooled testing method refers to mix multiple (ten or twenty) samples before testing. There is no infected individuals in the sample if the testing result is negative, while the further individual sample method is needed to identify the infected individual if the result is positive [11], and it was found that up to thirty samples pooled in a pool can increase test capacity with existing test resources and detect positive samples with sufficient diagnostic accuracy [12]. Obviously, the population-wide testing can help to quickly detect the infected individuals, including the asymptomatic infections, hence, it helps to quickly isolate the infections. Therefore, the large-scale population-wide screening program gradually becomes an effective intervention to rapidly contain the spread of epidemic during an outbreak response [13–15]. Actually, the implementation scheme of large-scale population-wide testing depends on the existing equipment and test kits. Considering the limitation of test resources, Hernandez and Valentinotti studied a optimal sample selection strategy which selected 5% or 10% of the whole population with excluding the sample of individuals who have a low probability of being infected (recently tested ones). Then under fixed numbers of tests, this strategy can effectively reduce the overall infected-person-days [16]. However, it should be mentioned that on the background of the COVID-19 local outbreaks in China, the nucleic acid test is implemented every one to three days of the whole population [17, 18]. For example, during the local outbreaks happening at Yangzhou (2021/7/28 to 2021/8/26) and Xi’an (2021/12/9 to 2022/1/9), the comprehensive and routine population wide screening strategy ensures the timeliness of early case detection and interruption of the spread of COVID-19, which plays critically important roles in the prevention and control strategy. However, the quantitative role of large-scale population-wide nucleic acid screening in mitigating the COVID-19 epidemics in China remains unclear.

Mathematical models are frequently used as the powerful tools to study the transmission dynamics of COVID-19 [1, 2], evaluate the effectiveness of control interventions [19, 20] or the mass vaccination program [21, 22]. Given the population-wide nucleic acid testing, the authors used a statistical model to study the optimal number of samples combined into a pool under different infection rates [11]. It should be mentioned that the population-wide nucleic acid testings are carried out every few days, which are actually the combination of the dynamic system and discrete events. In the existing modelling frameworks, fixed-moments impulsive differential equations were widely used in modelling the intermittent prevention and control measures for containing the spread of infectious diseases, and these models assumed that measures are carried out at fixed discrete times [23–26]. Therefore, the fixed-moments impulsive differential equations can be the potential modelling frameworks to describe the pulsed large-scale testing. To our best knowledge, few modelling studies quantitatively investigated the impact of pulsed population wide nucleic acid screening strategy on controlling and stopping the spread of COVID-19. Qualifying the issues through mathematical models follows the scope of this study.

The main purpose of this study is to propose the epidemic model considering nucleic acid screening, focus on exploring its role to contain the COVID-19 outbreaks, and providing the important guidance for the implementation of the large-scale population wide screening in controlling the future waves. The rest of the paper is organized as follows. In Methods section, we firstly extend the classic *SEIR* model characterizing the disease progress to a deterministic impulsive dynamical model to describe the transmission of COVID-19 with the pulse nucleic acid screening. The data and the fitting methods are also described in details, and we calibrate the model by fitting it to the epidemic data of the local outbreaks in Yangzhou city and Xi’an city, China. In Results section, we reveal the role of nucleic acid screening through the scenario analysis in reducing the cumulative cases and shortening the time period of lockdown. We further extend the original model to a pulse model with periodic screening, and discuss the impact of the large-scale screening on mitigating the epidemic and the runs on medical resources. In Discussion and conclusion section, we give some discussions and conclusions.

## Methods

### Model

**Fig. 1:**
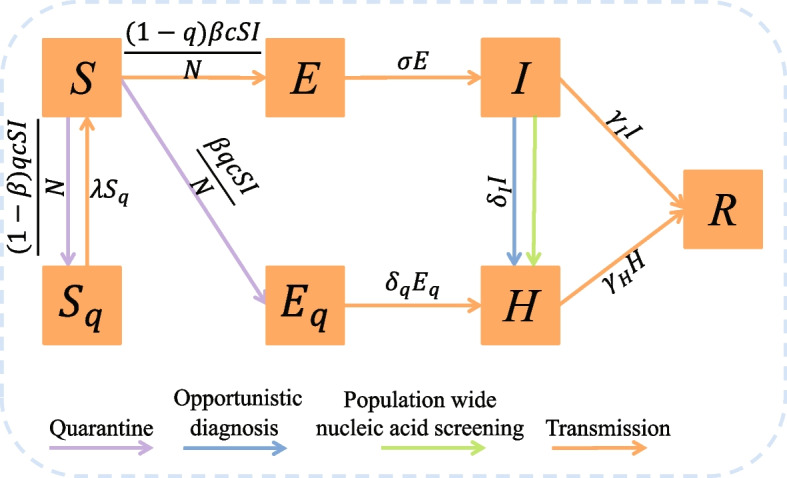
Schematic diagram of the model for illustrating the COVID-19 infection dynamics. The infected individuals in class *I* can be diagnosed and isolated through opportunistic diagnosis and the population wide nucleic acid screening

According to the epidemic status of COVID-19 infections and the implementation of control interventions, including the close contact tracing and quarantine and the nucleic acid screening adopted in the local outbreaks in Yangzhou and Xi’an, the total population *N* is divided into seven compartments: susceptible (*S*), exposed (*E*), infected (*I*), quarantined susceptible ($S_q$), quarantined exposed ($E_q$), confirmed and isolated (*H*) and recovered (*R*). It should be noticed that in our model framework, the infected population (*I*) will move into the confirmed and isolated class (*H*) through two ways: fever clinic (called opportunistic confirmed) and population wide nucleic acid screening. As we mentioned in the introduction, almost all the infections can be detected through the nucleic acid screening and then isolated, hence the asymptomatic compartment is not involved in our model. The transmission diagram is shown in Fig. 1.

Let $\tau _i$ denote the time when the infected individuals are confirmed and isolated through the population wide nucleic acid screening for the whole society, with $i=1,2,\cdots$ being the times implementing screening. We assume that this part of infected individuals can be diagnosed simultaneously, and immediately move to compartment *H* at $\tau _i^+$. Based on the above assumptions and previous studies, the transmission dynamics is governed by the following impulsive model:

1 $$\begin{aligned} \left\{ \begin{array}{lll} \left. \begin{array}{lll} S'&{}=&{}-\frac{\left( \beta +q(t)(1-\beta )\right) c(t)SI}{N}+\lambda S_q,\\ E'&{}=&{}\frac{(1-q(t))\beta c(t)SI}{N}-\sigma E,\\ I'&{}=&{}\sigma E-(\delta _I(t)+\gamma _I)I,\\ S_q'&{}=&{}\frac{(1-\beta )q(t)c(t)SI}{N}-\lambda S_q,\\ E_q'&{}=&{}\frac{\beta q(t)c(t)SI}{N}-\delta _q(t)E_q,\\ H'&{}=&{}\delta _I(t) I+\delta _q(t) E_q-\gamma _H H,\\ R'&{}=&{}\gamma _I I+\gamma _H H,\\ \end{array} \right\} ~t\ne \tau _i,\\ \left. \begin{array}{lll} S(\tau _i^+)&{}=&{}S(\tau _i),\\ E(\tau _i^+)&{}=&{}E(\tau _i),\\ I(\tau _i^+)&{}=&{}\text{ max }\{I(\tau _i)-c_i,0\},\\ S_q(\tau _i^+)&{}=&{}S_q(\tau _i),\\ E_q(\tau _i^+)&{}=&{}E_q(\tau _i),\\ H(\tau _i^+)&{}=&{}H(\tau _i)+\text{ min }\{I(\tau _i),c_i\},\\ R(\tau _i^+)&{}=&{}R(\tau _i),\\ \end{array} \right\} ~t=\tau _i.\\ \end{array} \right. \end{aligned}$$

Considering the enhanced control interventions by the government, we introduced four time-dependent parameters into the system (i.e. model (1)). With the implementation of contact tracing, a proportion of *q* of individuals exposed to the virus is quarantined. Let $\beta$ be the transmission probability and *c* be the contact rate, then the quarantined individuals can move into compartment $E_q$ (or $S_q$) at a rate of $\beta cq$ (or $(1-\beta )cq$) if they are effectively infected (or not effectively infected). While the other proportion, $1-q$, missed from the contact tracing, will move into the exposed compartment *E* at a rate of $\beta c(1-q)$ once effectively infected or stay in the susceptible compartment *S* otherwise. Moreover, as the implementation of lockdown, the contact rate *c*(*t*) should be a decreasing function of time *t* with the following form [27, 28]

$$\begin{aligned} c(t)=\left( c_0-c_b\right) e^{-r_c t}+c_b, \end{aligned}$$

where $c_0$ is the contact rate before lockdown (i.e., before 2021/7/31 in Yangzhou and 2021/12/23 in Xi’an, respectively), $c_b$ is the minimum contact rate, and $r_c$ is the corresponding exponential decreasing rate of the contact rate. Similarly, we set the quarantined rate *q*(*t*) to be an increasing function of time *t* after lockdown, which takes the following form

$$\begin{aligned} q(t)=\left( q_0-q_b\right) e^{-r_q t}+q_b, \end{aligned}$$

where $q_0$ is the quarantine rate before lockdown, $q_b$ is the maximum quarantine rate, and $r_q$ is the exponential increasing rate of the quarantine rate.

In addition, the rate of diagnosis of infected individuals and quarantined exposed individuals in Xi’an are set to be piecewise functions [29], and the basis of function segmentation is consistent with the time of lockdown strategy [30], which are given by

$$\begin{aligned} \delta _I(t)=\left\{ \begin{array}{ll} \delta _{I0},&{}~\text{ before }~ \text{2021/12/23},\\ \delta _{Ib},&{}~\text{ after }~ \text{2021/12/23},\\ \end{array} \right. \end{aligned}$$

and

$$\begin{aligned} \delta _q(t)=\left\{ \begin{array}{ll} \delta _{q0},&{}~\text{ before }~ \text{2021/12/23},\\ \delta _{qb},&{}~\text{ after }~ \text{2021/12/23}.\\ \end{array} \right. \end{aligned}$$

We note that $c_i$ denotes the number of diagnosed individuals due to the *i*-th time population wide pooled nucleic acid testing during the local outbreaks. Then the term $\text{ min }\{I(\tau _i),c_i\}$ describes the newly confirmed and hospitalized individuals through the nucleic acid screening at time $\tau _i^+$, and the term $\text{ max }\{I(\tau _i)-c_i,0\}$ denotes the number of infected individuals who still remain in compartment *I* at time $\tau _i^+$.

### Data

**Fig. 2:**
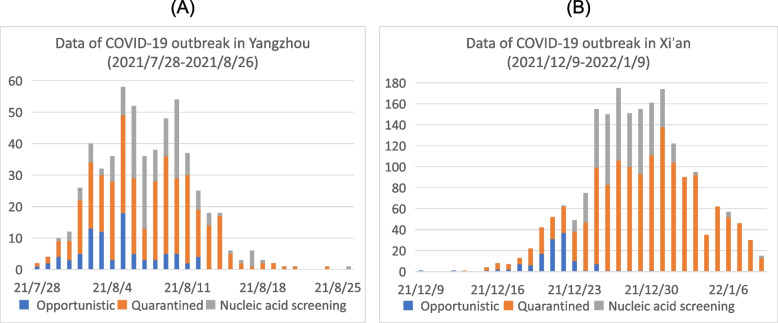
**A** Data of COVID-19 outbreak in Yangzhou from July 28, 2021 to August 26, 2021; **B** Data of COVID-19 outbreak in Xi’an from December 9, 2021 to January 9, 2022. The data include daily opportunistic confirmed cases, daily confirmed quarantined exposed cases and daily confirmed cases through nucleic acid screening

We obtained the epidemic data related to the local COVID-19 outbreaks in Yangzhou city of Jiangsu Province of China during 2021/7/28 to 2021/8/26 and Xi’an city of Shaanxi Province of China during 2021/12/9 to 2022/1/9 from the Health Commission of Jiangsu Province and the Health Commission of Shaanxi Province [31, 32], respectively. The data information includes the time series of the daily opportunistically diagnosed cases, daily confirmed cases from the quarantined population, and the daily diagnosed cases through the population wide nucleic acid screening, as shown in Fig. 2. It should be mentioned that the infected individuals can be diagnosed through opportunistic diagnosis such as fever clinic and population wide nucleic acid screening.

### Model fitting process

When fitting model (1) to the epidemic data of Yangzhou and Xi’an, we initially informated several parameters and initial conditions from the database and literuture [33–36]. This includes latent period ($1/\sigma$), the releasing rate of quarantined susceptible individuals ($\lambda$), initial susceptible (*S*(0)), quarantined susceptible ($S_q(0)$), quarantined exposed ($E_q(0)$), confirmed and isolated (*H*(0)), and recovered (*R*(0)) populations, as listed in Table 1. As mentioned in the introduction, the COVID-19 epidemic pattern in China is that each outbreak is an independent outbreak with a starting time and end time of confirm cases. Incorporating the public data, we set the initial conditions of $S_q(0)$, $E_q(0)$, *H*(0) and *R*(0) as 0, 0, 2, 0 in Yangzhou, and as 0, 0, 1, 0 in Xi’an, respectively.

**Table 1:** Definitions and values of parameters and variables in Yangzhou and Xi’an

		Definition	Mean value (std)		Source
			**Yangzhou**	**Xi’an**	
Parameter					
*c*(*t*)	$c_0$	Contact rate before lockdown	22.90(5.44)	24.70(6.33)	Estimated
	$c_b$	Minimum contact rate with control strategies	3.31(0.97)	2.38(1.83)	Estimated
	$r_c$	Exponential decreasing rate of contact rate	1.12(0.48)	0.32(0.24)	Estimated
$\beta$		Transmission probability from *I* to *S* per contact	0.09(0.02)	0.06(0.02)	Estimated
*q*(*t*)	$q_0$	Quarantined rate before lockdown	0.23(0.06)	0.25(0.05)	Estimated
	$q_b$	Maximum quarantined rate with control strategies	0.88(0.03)	0.86(0.11)	Estimated
	$r_q$	Exponential increasing rate of quarantined rate	0.61(0.21)	0.78(0.98)	Estimated
$\lambda$		Releasing rate of quarantined susceptibles	1/14	1/14	[34, 35]
$\sigma$		Transition rate of exposed individuals to the infected class	1/3	1/3	[36]
$\delta _I(t)$	$\delta _{I0}$	Diagnose rate of infected individuals before lockdown	0.10(0.02)	0.19(0.05)	Estimated
	$\delta _{Ib}$	Diagnose rate of infected individuals after lockdown	–	0.02(0.01)	Estimated
$\delta _q(t)$	$\delta _{q0}$	Diagnose rate of quarantined individuals before lockdown	0.23(0.02)	0.27(0.08)	Estimated
	$\delta _{qb}$	Diagnose rate of quarantined individuals after lockdown	–	0.19(0.05)	Estimated
$\gamma _I$		Recovery rate of infected individuals	0.04(0.01)	0.11(0.06)	Estimated
$\gamma _H$		Recovery rate of confirmed and isolated infections	0.12(0.0003)	0.12(0.005)	Estimated
Variable					
*S*		Susceptible population	$1.70\times 10^6$	$1.29\times 10^7$	[33]
*E*		Exposed population	36.19(15.73)	16.41(7.61)	Estimated
*I*		Infected population	18.31(2.94)	1.03(0.27)	Estimated
$S_q$		Quarantined susceptible population	0	0	Data
$E_q$		Quarantined exposed population	0	0	Data
*H*		Confirmed and isolated population	2	1	Data
*R*		Recovered population	0	0	Data

By assuming that each data point follows a Poisson distribution with a mean assumed as the observed counts, we sample 200 data of each time point from the corresponding distributions, consequently, we obtain 200 time series data of daily opportunistically confirmed cases, daily confirmed cases from quarantined population and the cumulative confirmed cases [29, 37]. Then, for each sampled time series, we use the least squared method to simultaneously fit the daily opportunistically confirmed cases, daily confirmed cases from quarantined population and the cumulative confirmed cases, where a *priori* distribution for each unknown parameter and initial condition is provided. Here, the ODE system is solved by the “ODE45” function while the “fmincon” function is used to search the optimal solutions of the objective function. The objective function is defined as the residual sum of squares between the real data of the time series of the opportunistic daily confirmed cases, daily confirmed cases from the quarantined population and the cumulative confirmed cases and the correspondingly predicted numbers by solving system (1). Therefore, we obtain 200 outputs of the unknown parameters and initial conditions, from which we can obtain their means. Also, we then calculate the 2.5% and 97.5% percentiles of the 200 solutions to generate the 95% confidence interval (CI) for quantile of the fitting results.

## Results

### Model fitting results and senario analysis

**Fig. 3:**
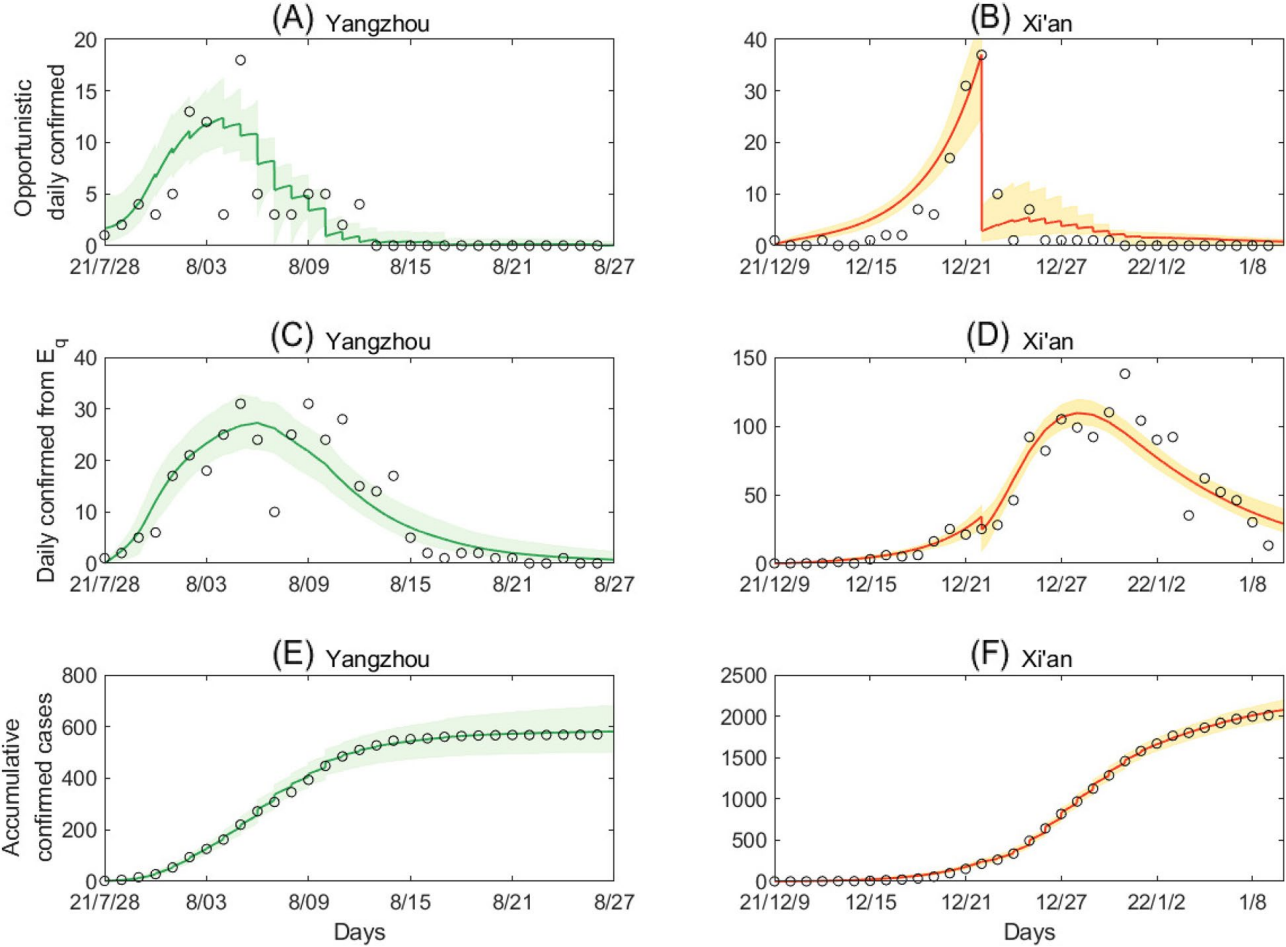
Best model fitting results for the transmission dynamic model in Yangzhou and Xi’an. **A**, **C** and **E** are the daily opportunistic confirmed cases from *I*, the daily confirmed cases from $E_q$ and the cumulative confirmed cases in Yangzhou, respectively. Similarly, **B**, **D** and **F** are those in Xi’an. The yellow and green curves are the estimated curves in Yangzhou and Xi’an, respectively, with the shadow areas being the corresponding 95% confidence intervals. The circles are the corresponding observed data

By fitting model (1) to daily opportunistically confirmed cases, daily confirmed cases from quarantined population and the cumulative confirmed cases simultaneously, we showed the fitting results of the local outbreaks in Xi’an and Yangzhou in Fig. 3. Based on the fitting results, we obtained the mean and the standard diviation of the unknown parameters and initial conditions, as listed in Table 1. Further, to test if the key parameters correlate to each other, we yield the symmetric covariance matrix of seven parameters $\beta$, $c_0$, $q_0$, $\gamma _I$, $\delta _{I_0}$, $\delta _{q_0}$ and $\gamma _H$ estimated by fitting the data of Xi’an in Table 2. It follows from Table 2 that the estimated values of the initial contact rate ($c_0$) and the recovery rate $\gamma _I$ are of the biggest positive correlation coefficient. It highlights the importance to use the least squared methods with a *priori* distribution to each parameter for including the useful information of the parameters and to fix them into a reasonable range.

**Table 2:** The symmetric covariance matrix of parameters $\beta$, $c_0$, $q_0$, $\gamma _I$, $\delta _{I_0}$, $\delta _{q_0}$ and $\gamma _H$ in Xi’an

	$\beta$	$c_0$	$q_0$	$\gamma _I$	$\delta _{I_0}$	$\delta _{q_0}$	$\gamma _H$
$\beta$	0.0005	-0.1002	0.0005	-0.0007	0.0007	-0.0001	-0.0000006
$c_0$		40.3123	-0.1610	0.2988	-0.0092	0.1101	-0.0016
$q_0$			0.0025	-0.0019	0.0006	-0.0027	-0.000005
$\gamma _I$				0.0036	-0.0005	0.0008	0.000007
$\delta _{I_0}$					0.0028	0.0005	-0.00001
$\delta _{q_0}$						0.0076	-0.00003
$\gamma _H$							0.00003

**Fig. 4:**
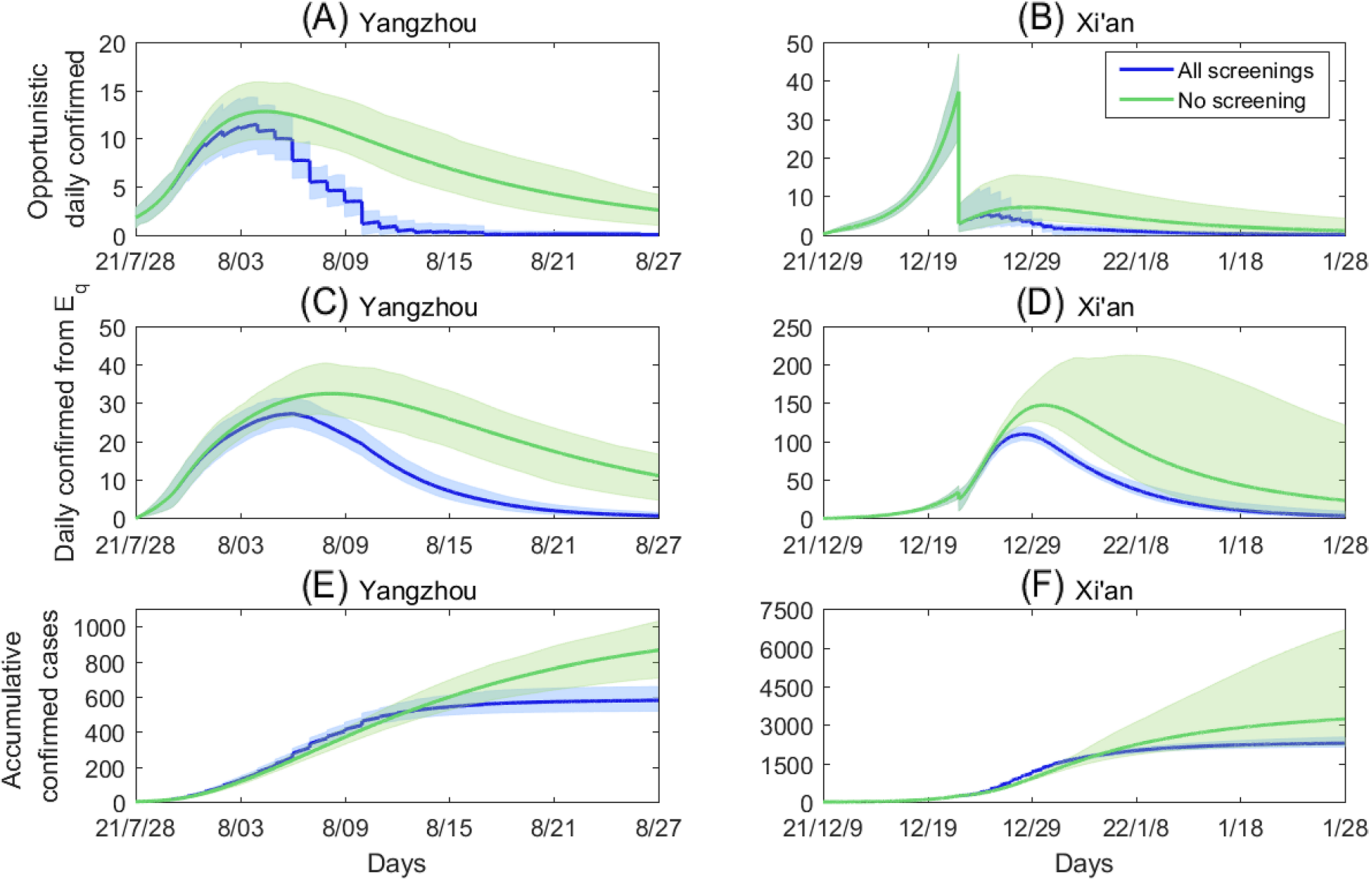
Comparing the impact of previous implemented screenings and implementing no screening on the COVID-19 epidemic in Yangzhou and Xi’an. The blue and green curves are the estimated curves corresponding to previous screening and no screening strategies, respectively, with the shadow areas being the corresponding 95% confidence intervals. The other parameter values are fixed as those listed in Table 1

In this subsection, we investigate the impact of population wide nucleic acid screening on controlling the outbreak of COVID-19 and further discuss how the frequency and time of implementing population wide nucleic acid screening affect the spread of COVID-19 and the case clearing time. Firstly, we compared the previous screening scheme and no screening administered in Yangzhou and Xi’an, and plotted the daily number of opportunistic confirmed cases, the confirmed cases from $E_q$ and the cumulative number of confirmed cases under the above two cases, shown in Fig. 4. It follows from Fig. 4 that compared to the previous screening strategy, the daily confirmed number of infected cases from *I* through opportunistic diagnosis and quarantined exposed cases from $E_q$, and the cumulative number of confirmed cases remarkably increase both in Yangzhou and Xi’an without population wide nucleic acid screening strategy. To be specific, the cumulative number of confirmed cases will increase by $77.7\%$ in Yangzhou and $62.2\%$ in Xi’an without implementing population wide nucleic acid screening, shown in Table 3. These results indicate that previous screening strategy can effectively reduce the final epidemic size of infections, which play an important role in containing the spread of COVID-19.

**Table 3:** The impact of nucleic acid screening scenarios on the number of cumulative confirmed cases

Screening scenario	Accumulative confirmed cases	
	Yangzhou	Xi’an
Current screening program	584	2288
The first half screenings in Yangzhou (Screening before 22/1/1 in Xi’an)	752(+28.8%)	2380(+4.0%)
Screening every other two days	841(+44.0%)	2999(+31.1%)
The last half screenings in Yangzhou (Screening after 22/1/1 in Xi’an)	853(+46.1%)	3651(+59.6%)
No screening	1038(+77.7%)	3712(+62.2%)

**Fig. 5:**
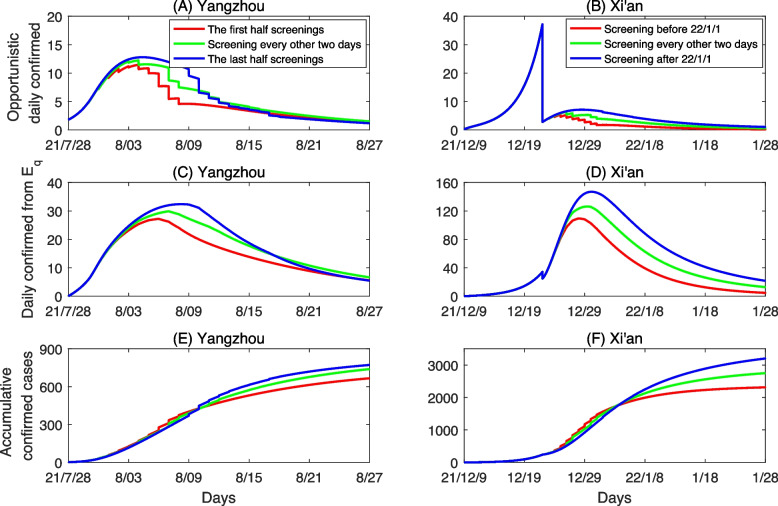
The impacts of various nucleic acid screening schemes on the COVID-19 epidemic in Yangzhou and Xi’an. The specific schemes are: Remain the first half screenings in Yangzhou (remain the screenings before 22/1/1 in Xi’an); Remain the screenings every other two days; Remain the last half screenings in Yangzhou (remain the screenings after 22/1/1 in Xi’an). The other parameter values are fixed as those listed in Table 1

Then we focus on discussing the effectiveness of three different screening schemes: In the first scheme, we assume that the first half screenings of the previous screening strategy are remained while the last half screenings will not be carried out for Yangzhou (i.e. the screenings before 2022/1/1 of the previous screening strategy are remained while the screenings after 2022/1/1 will not be carried out for Xi’an); In the second scheme, we assume that one cycle screening is implemented every other two days; In the third scheme, we remain the last half screenings of the previous screening strategy while the first half screenings will not be administered for Yangzhou (i.e. remaining screenings after 2022/1/1 of the previous screening strategy while the screenings before 2022/1/1 will not be administered for Xi’an). Under different screening schemes, we illustrate the impacts of various screening strategies on the COVID-19 epidemic in Yangzhou and Xi’an in Fig. 5. Among the above three screening strategy, we find that the first scheme results in the most significant decrease of the cumulative number of confirmed cases while the third scheme has little effect on reducing the cumulative number of confirmed cases both in Yangzhou and Xi’an. We further compared the cumulative number of confirmed cases according to different screening schemes in Table 3. It can be observed that when reducing the times of population wide nucleic acid screening, the earlier the nucleic acid screening, the impact on controlling the spread of COVID-19 is more remarkable. However, the nucleic acid screening in the late stage of the epidemic is of little help to mitigate the outbreak of COVID-19.

The above analysis quantitatively showed how the population wide necleic acid screening can help to stop the spread of COVID-19 when we aimed at controlling the cases into zero quickly. In the next section, we focus on discussing the role of nucleic acid screening in mitigating the COVID-19 when we aimed at controlling the epidemics in a low level. Correspondingly, we assume that the lockdown strategy would be released.

### Impacts of the screening on mitigating COVID-19 outbreaks and the runs on medical resource

In this section, we focus on investigating how alternative population wide screening program affects the transmission dynamics of COVID-19, especially, analysing the impacts of the screenings on the runs on medical resources. To this end, we assume to screening the population using nucleic acid test at a constant rate $q_s$. That is, a constant ratio of infected population (class *I*) will be tested at the screening time. In addition, we set $d_e$ as the detection efficiency rate of the screening, hence, $d_eq_s$ denotes the effective screening rate. The periodical screening strategy is considered with a period of $T_s$ (days), that is, every $T_s$ days, we carry out one cycle of the population wide nucleic acid screening with the ratio $d_eq_s$ of infected individuals being confirmed and isolated.

2 $$\begin{aligned} \left\{ \begin{array}{lll} \left. \begin{array}{lll} S'&{}=&{}-\frac{\left( \beta +q(t)(1-\beta )\right) c(t)SI}{N}+\lambda S_q,\\ E'&{}=&{}\frac{(1-q(t))\beta c(t)SI}{N}-\sigma E,\\ I'&{}=&{}\sigma E-(\delta _I(t)+\gamma _I)I,\\ S_q'&{}=&{}\frac{(1-\beta )q(t)c(t)SI}{N}-\lambda S_q,\\ E_q'&{}=&{}\frac{\beta q(t)c(t)SI}{N}-\delta _q(t)E_q,\\ H'&{}=&{}\delta _I(t) I+\delta _q(t) E_q-\gamma _H H,\\ R'&{}=&{}\gamma _I I+\gamma _H H,\\ \end{array} \right\} ~t\ne nT_s,n=1,2,\cdots ,\\ \left. \begin{array}{lll} I(nT_s^+)&{}=&{}(1-d_eq_s)I(nT_s),\\ H(nT_s^+)&{}=&{}H(nT_s)+d_eq_sI(nT_s),\\ \end{array} \right\} ~t=nT_s,n=1,2,\cdots .\\ \end{array} \right. \end{aligned}$$

As we mentioned above, the main purpose in this section is to analyze the influence of screening in mitigating the COVID-19 epidemics, especially in avoiding the runs on medical resources. That is to say, we would not tend to adopt such a strict control strategy by locking down the city. Correspondingly, the contact rate will not decreased to $c_b$, instead, it can vary with the range $[c_b,c_0]$. The other parameter values are fixed as those estimated from the outbreak of Xi’an, as listed in Table 1.

**Fig. 6:**
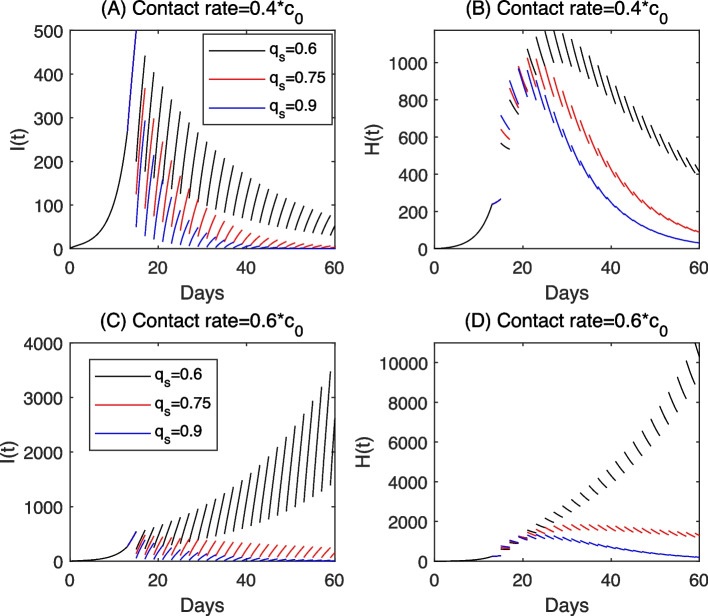
Solutions of model (2). Here, we assumed a screening frequency of each two days (i.e. $T_s=2$). $d_e=1$ and the other parameter values are fixed as the same as those estimated from the outbreak in Xi’an, as listed in Table 1

Based on the above assumptions, by varying the contact rate and the screening rate, we plotted the infected population (*I*(*t*)) and confirmed and isolated population (*H*(*t*)) in Fig. 6. In Fig. 6(A-B), fixing the parameters and initial conditions as the same as those related to the outbreak of Xi’an and increasing the contact rate to $0.4c_0$ after Dec. 23, 2021, we present the impacts of the intensive screening program (one cycle every two days) on the transmission dynamics. In this circumstance, the outbreak in Xi’an is under control as the infected cases persistently decrease to a very low level in the 60 days. As we further increase the contact rate to $0.6c_0$, if the screening rate is high enough ($q_s=0.75$ or $q_s=0.9$), the outbreak is still under control. However, the screening program seems not to be able to control the outbreak if the screening rate is only 0.6 (the black curves in Fig. 6(C-D)).

**Fig. 7:**
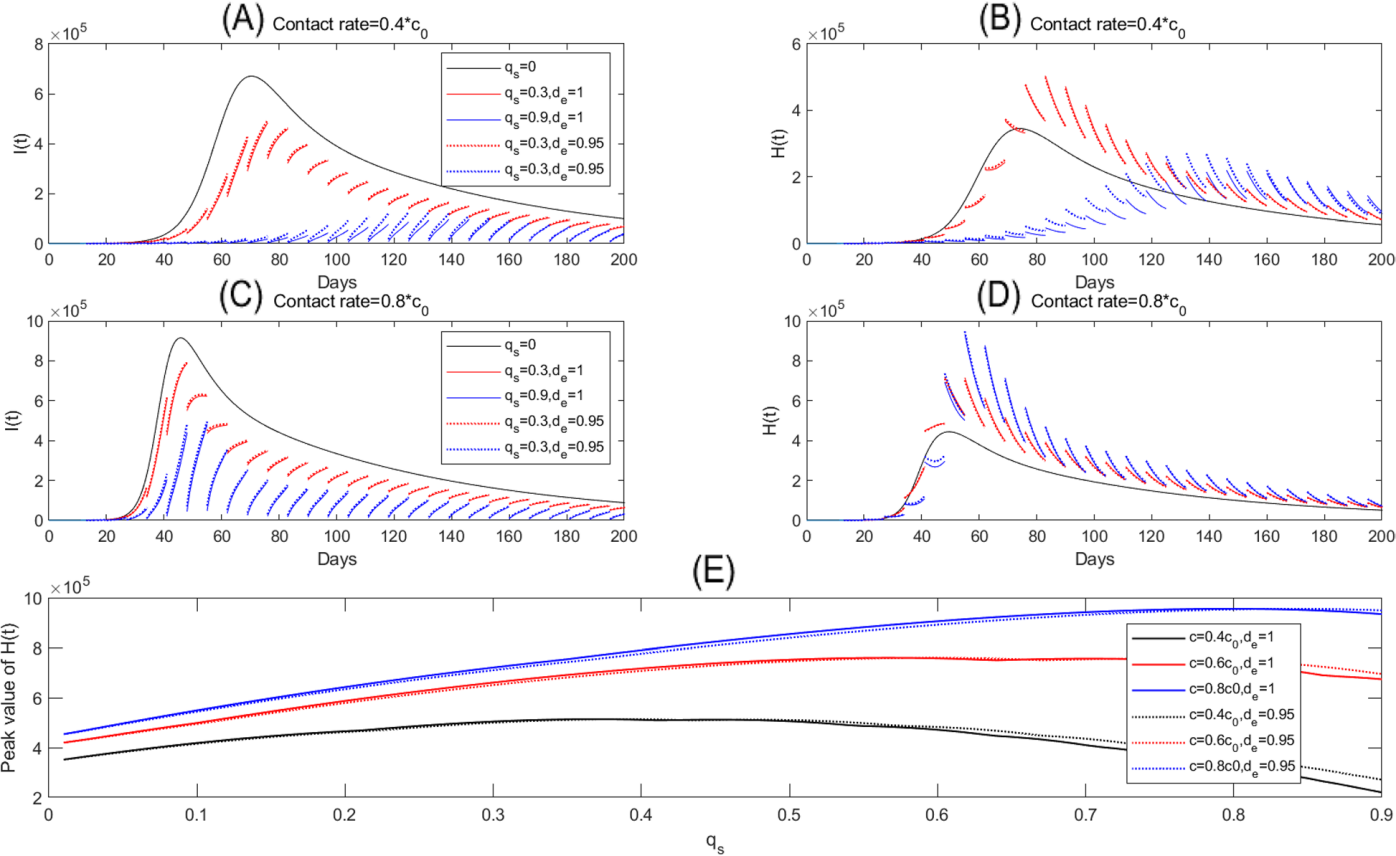
**A**-**D** Solutions of model (2); **E** Relation curves of the peak values of confirmed and isolated population (*H*(*t*)) with respect to the screening rate. Here, we fix the screening period as $T_s=7$, and vary the screening rate and contact rate. The other parameters are fixed as the same as those estimated from the outbreak in Xi’an

In Fig. 7, in addition to release the strict NPIs by setting a higher contact rate, we also released the intensive screening by fixing a lower screening frequency. Definitely, there can be a large outbreak in this case, hence we move our focus to avoid the runs on medical resource instead of control the cases into zero. With such a purpose, we should control the peak of the confirmed and isolated population, with a ratio of them being hospitalized, below a critical value. In this sense, we observed the paradox phenomenon of the screening rate in Fig. 7(A-B), that is, the peak value of *H*(*t*) for $q_s=0.3$ is higher than those for $q_s=0$ and $q_s=0.9$. In Fig. 7(E), we showed in more details that when the screening rate is small, the peak value of the hospitalized population increases as the screening rate increases. This indicates that the screening program may aggravate the runs on medical resources when the screening rate in a certain range. Comparing Fig. 7(A-B) with (C-D), we find that when the contact rate is bigger, the screening program can lead to a higher peak value of hospitalized population in a larger range of the screening rate, which can be also clearly seen from Fig. 7(E). Moreover, when decrease the detection efficiency $d_e$ from 1 to 0.95 in Fig. 7, we find that the soluations of *I*(*t*), *H*(*t*) are very close to each other by comparing the dashed curves and the solid curves with a same color. Also, the change rule of peak value of *H*(*t*) with respect to parameter $q_s$ seems to have a similar trend, particularly in terms of the existence of the paradox. This implies that the results are robust with a small change to parameter $d_e$.

**Fig. 8:**
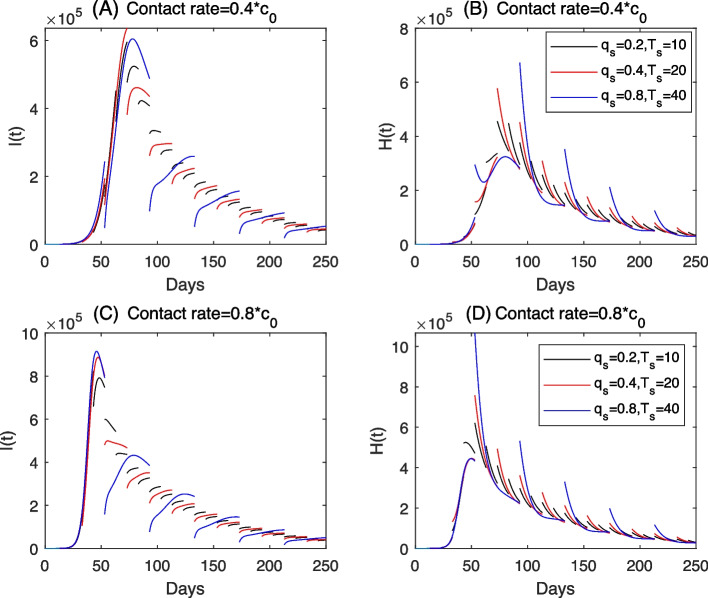
Solutions of model (2) by choosing different combinations of the screening rate and the screening period. Here $d_e=1$ and the other parameters are fixed as the same as those estimated from the COVID-19 outbreak in Xi’an

Figure 7 shows an important fact that there exists the potential risk of runs on medical resources to use the control measure of population wide screening when the COVID-19 outbreaks are in high epidemic level. For this reason, instead of testing the whole population each time, an alternative strategy by testing a relatively small population each time but with a high screening frequency may be a better choice to balance the control of COVID-19 epidemics and the runs on medical resources. Note that, due to the testing capacity, the screening ratio $q_s$ is usually negatively correlated to the screening frequency, that is, a longer sreening period $T_s$ means a higher screening during each screening period and vice versa. For this reason, we assume that the screening period $T_s$ is proportional to the screening rate $q_s$ with a constant coefficient. And we consider different combinations of $T_s$ and $q_s$ under this assumption, i.e. initially set $T_s=10$ and $q_s=0.2$, then double $T_s$ to $T_s=20$ and further to $T_s=40$, correspondingly double $q_s$ to $q_s=0.4$ and futher to $q_s=0.8$. The corresponding results of *I*(*t*) and *H*(*t*) are plotted in Fig. 8. It’s easy to see from Fig. 8 that the peak value of the hospitalized population can be smaller for the combination of a shorter screening period $T_s$ and a lower screening ratio $q_s$. This also supports that to avoid the runs on medical resource, we should try to adopt a screening program which screens a lower population each time with a higher frequency when the outbreak is in a high epidemic level already.

## Discussion and conclusion

In this study, we proposed a mathematical model with pulse population-wide nucleic acid screening, which is a main part of the zero-COVID policy (i.e. a policy to control the cases into zero in a short period). The model was calibrated by fitting the epidemic data related to the local COVID-19 outbreaks in Yangzhou city of Jiangsu province and Xi’an city of Shaanxi province, China. With the proposed models, we focus on investigating two essential issues: a) the role of the large-scale screening program in the zero-COVID policy; b) the impact of the large-scale screening on the transmission dynamics of COVID-19 and the runs on medical resources.

To analyze the impact of the large-scale population wide nucleic acid screening on reaching the goal of the zero-COVID policy, we conducted senario analysis by deleting part times of the real screening program in Yangzhou and Xi’an. In line with the goal of the zero-COVID policy, we compared the final size and the time that the reported cases reach a certain low level (threshold value of one case for example) in different schedules of the screening program. We showed that the cumulative confirmed cases increase by $77.7\%$ in Yangzhou and $62.2\%$ in Xi’an if we totally cancel the screening program during the outbreaks. In addition, cancelling the large scale screening program can postpone the time of reopening for more than one month. Considering to delete a fixed times of the screening in Yangzhou and Xi’an, we found that the screening, at the time period that the outbreak was controlled to a low level or in the decreasing phase already, has limited influence on the epidemics. In other words, screening earlier could be more cost-effectiveness to balance the control of the outbreak and the costs of the testing.

Considering a periodic screening strategy, we extended our original model to a mathematical model with pulse screening at the fixed time points. The result, from the simulations by keeping the intensive screening and increasing the contact rate, shows that the intensive screening program can provide a certain space to release the highly strong NPIs. In other words, given the cost-effectiveness, we may not need to lockdown the city even though the goal is to control the cases into zero in a short period. On the other hand, if we switch our goal to mitigate the epidemics instead of stopping its spread by the control interventions, we could release the highly strict interventions, including the NPIs and the intensive screening. It’s easy to image that the large-scale screening will lead to a large ratio of infected population be diagnosed instantaneously, consequently, a large population need to be hospitalized when the new wave is in a high epidemic level. Therefore, there exists the potential risk of the runs on medical resource induced by the large-scale screening. This is true and we observed the paradox phenomenon of the large-scale screening in the sense of runs on medical resource, as shown in Fig. 7. The driver of the paradox phenomenon should be that the screening is not strong enough, corresponding to a limited screening rate, to drop the epidemic into a low level. This indicates that the population-wide nucleic acid screening can have very limited effects on controlling the epidemics of COVID-19 if the outbreak is in a relatively high epidemic level or there has already been runs on medical resources. Given the runs of the medical resource, we further showed that if we try to screen a fixed number of the population, a higher frequency by screening a smaller population in each time can be a better choice to balance the control of the epidemics and the runs on the medical resources. It should be noticed that our study bases on the whole population nucleic acid tests of the COVID-19 local outbreaks in China. For low-income countries or those countries with limited test resources, optimal sample selection strategy with selecting a proportion of the whole population and excluding the samples of individuals who have a low probability of being infected (recently tested ones) should be explored, which can be further studied in the future work.

## Data Availability

Data and relevant code for this research work are stored in GitHub: https://github.com/Qian199210/nucleic-acid-screening.git.

## Abbreviations

SARS-CoV-2: Severe Acute Respiratory Syndrome coronavirus type 2
COVID-19: Coronavirus disease 2019
NPIs: Non-pharmaceutical interventions
ODE: Ordinary differential equation
CI: Confidence interval
